# Gender inequalities in secondary prevention of cardiovascular disease: a scoping review

**DOI:** 10.1186/s12939-024-02230-3

**Published:** 2024-07-23

**Authors:** Irene López Ferreruela, Blanca Obón Azuara, Sara Malo Fumanal, María José Rabanaque Hernández, Isabel Aguilar-Palacio

**Affiliations:** 1 Internal Medicine Service, Miguel Servet University Hospital, Saragossa, Spain; 2 Intensive Medicine Service, Lozano Blesa University Hospital, Saragossa, Spain; 3 GRISSA Research Group. IIS Aragón, Aragon Health Sciences Institute, Saragossa, Spain; 4 Faculty of Medicine, University of Zaragoza, Saragossa, Spain

**Keywords:** Gender inequalities, Cardiovascular disease, MACE, Secondary prevention, Scoping review

## Abstract

**Background:**

Despite significant progress in cardiovascular disease (CVD) management, it remains a public health priority and a global challenge. Within the disease process, health care after a cardiovascular event (secondary prevention) is essential to prevent recurrences. Nonetheless, evidence has suggested the existence of gender disparities in CVD management, leaving women in a vulnerable situation. The objective of this study is to identify all available evidence on the existence of gender differences in health care attention after a major adverse cardiovascular event.

**Methods:**

A scoping review following the structure of PRISMA-ScR was conducted. To define the inclusion criteria, we used Joanna Briggs Institute (JBI) population, concept, context framework for scoping reviews. A systematic search was performed in MEDLINE (PubMed), EMBASE and Cochrane. The methods of this review are registered in the International Platform of Registered Systematic Review and Meta-Analysis Protocols (INPLASY) (INPLASY202350084).

**Results:**

The initial search retrieved 3,322 studies. 26 articles were identified manually. After the reviewing process, 93 articles were finally included. The main intervention studied was the pharmacological treatment received (*n* = 61, 66%), distantly followed by guideline-recommended care (*n* = 26, 28%) and cardiac rehabilitation (CR) referral (*n* = 16)”. Literature described gender differences in care and management of secondary prevention of CVD. Women were less frequently treated with guideline-recommended medications and seem more likely to be non-adherent. When analysing guideline recommendations, women were more likely to make dietary changes, however, men were more likely to increase physical activity. Studies also showed that women had lower rates of risk factor testing and cholesterol goals attainment. Female sex was associated with lower rates of cardiac rehabilitation referral and participation.

**Conclusions:**

This review allowed us to compile knowledge on the existence of gender inequalities on the secondary prevention of CVD. Additional research is required to delve into various factors influencing therapeutic disparities, referral and non-participation in CR programs, among other aspects, in order to improve existing knowledge about the management and treatment of CVD in men and women. This approach is crucial to ensure the most equitable and effective attention to this issue.

**Supplementary Information:**

The online version contains supplementary material available at 10.1186/s12939-024-02230-3.

## Background

Cardiovascular disease (CVD), refers to a group of medical disorders that affect the heart and blood vessels and cause a variety of health problems, such as coronary heart disease, cerebrovascular disease, or peripheral arterial disease [1]. The etiology of CVD may be due to multiple factors and its increasing incidence is associated with a high frequency of individual risk factors, such as high blood pressure (BP), smoking, and obesity. Environmental determinants, demographic changes towards an aging population, the appearance of risk factors in developing countries and socioeconomic variables, among others, also play and important role in the incidence of CVD [2].

Despite significant progress in the prevention and treatment of CVD, it remains a public health priority and a global health challenge. It is the leading cause of morbidity and mortality in Europe and worldwide. In 2020, CVD accounted 1.70 million deaths in the European Union, 36% of all deaths and about 20% of all premature deaths (before the age of 65) [3, 4].

Within the disease process, health care after a cardiovascular event (so-called secondary prevention) is essential to reduce mortality and prevent recurrences [5]. This secondary CVD prevention involves a combination of elements such as lifestyle changes to minimize risk factors (e.g. increased daily activity and smoking cessation), adherence to cardioprotective medication, participation in cardiac rehabilitation (CR) programs and follow-up visits to monitor and control certain parameters (cholesterol, BP, body mass index or BMI) by healthcare professionals [6, 7].

The World Health Organization uses the term “gender medicine” to refer to the study of how biological (sex-based) and socio-economic and cultural (gender-based) differences influence people's health. [8, 9]. “Gender medicine” is the study of how diseases differ between men and women in terms of prevention, clinical manifestation, diagnostic and therapeutic approaches, prognosis, psychosocial effects and interactions with the health care system [10]. Regarding CVD management, evidence has suggested the existence of gender disparities, leaving women in a vulnerable situation [5, 11]. However, while this has been widely described for primary prevention and hospital care during the cardiac event [12, 13], gender inequalities in secondary prevention are much less well known.

Therefore, the objective of this study is to identify and understand the best evidence on the existence of gender differences in health care attention after a major adverse cardiovascular event (MACE). The results of this comprehensive review hold the potential to fill knowledge gaps and provide valuable information to address gender inequalities in the field of secondary CVD prevention.

## Methods

### Review of the literature

This scoping review was developed following the guideline structure of preferred reporting items for systematic reviews and meta-analyses extension for scoping reviews statement (PRISMA- ScR’s) [14]. The methods were specified and registered in the International Platform of Registered Systematic Review and Meta-Analysis Protocols (INPLASY) on 23 May 2023 (registration number INPLASY202350084) [15].

### Eligibility criteria

To define the inclusion criteria, we used Joanna Briggs Institute (JBI) population, concept, context framework for scoping reviews [16]. So, our population was the published literature related to secondary prevention in patients who have suffered a MACE. We defined MACE as those people with myocardial infarction, acute coronary syndrome, or stroke. These diagnoses correspond to codes I21 and I60-I63 of the International Classification of Diseases, 10th revision (ICD-10). Secondary CVD prevention can be understood as any strategy aimed to prevent a recurrent MACE and reduce the likelihood of a first MACE in patients with known CVD, including coronary artery disease, cerebrovascular arterial disease, peripheral arterial disease, and atherosclerotic aortic disease. The main secondary prevention strategies studied were: pharmacological treatment; cardiac rehabilitation (CR) programs, defined as a medically supervised program designed to improve cardiovascular health after CVD, including exercise counselling and training, education on heart-healthy lifestyle and risk factor control; health system follow-ups; and guideline-recommended lifestyle counselling. The concept of this scoping review are studies about gender inequalities, as the unfair and preventable differences that exist between women and men in the state of health care. Finally, the context is focused on the health services setting and, in particular, on the secondary CVD prevention measures, such as disease management, pharmacological treatment regimen, adherence, rehabilitation, access to services, visits to general practitioner and nurse and referral to specialists.

We included studies of any design, setting or duration if they reported gender inequalities in secondary prevention on patients with MACE. Articles in English were selected because it is the language in which most of the publications are written, in addition to Spanish, which is the native language of the authors. We restricted the search to articles from the last ten years (2014 to 2023) in order to explore MACE’s secondary prevention intervention related to the latest guideline recommendations.

Studies that focused on pathologies other than MACE, addressed primary prevention, treatment of CVD risk factors or explored medical care during the event were excluded. We also excluded publications that analysed differences due to biology, e.g., different pharmacological effectiveness due to biological factors associated with sex. We excluded those publications that focused on health outcomes instead of health care, such as quality of life or functionality. Finally, short communications (conference abstracts or posters) without full text available were not included.

### Search strategy and study selection

Three concepts combined were used to develop our search strategy: (1) MACE, (2) gender disparities, and (3) secondary prevention measures.

Extensive literature searches were conducted in MedLine (via Pubmed), Cochrane and Embase, from inception to 20 February 2023 with some restrictions (abstract available, last ten years, adults only and language: English and Spanish).

Searches were piloted in MedLine (Pubmed) and then adapted to run in the other databases assisted by the Polyglot Search Translator [17]. A manual or snowball search was also conducted to identify other relevant studies in additional sources.

**Fig. 1:**
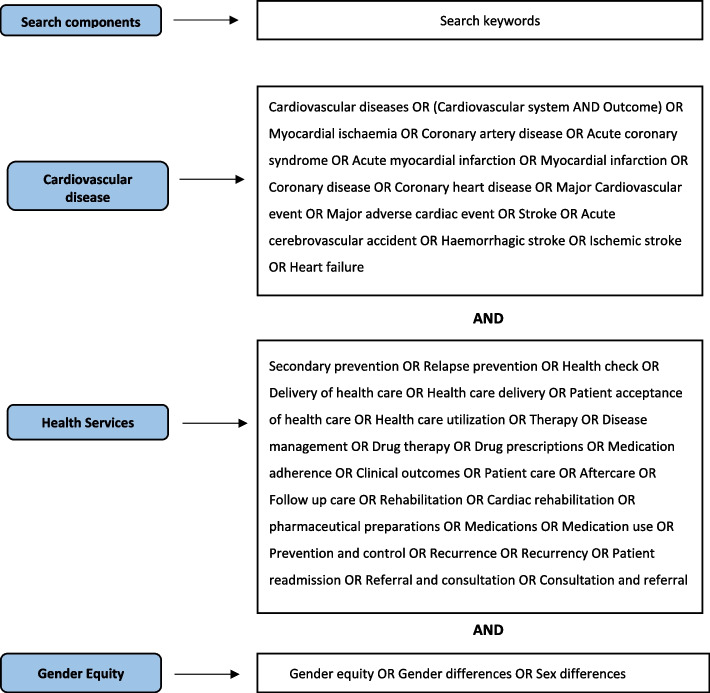
Search terms

The complete search strategy is shown in the Supplementary material Additional file 1. Search terms used are shown in Fig. 1.

Study screening and selection was carried out in different phases. First, duplicate studies were automatically deleted by the online software CONVIDENCE [18] and manually with RAYYAN [19]. Secondly, potentially relevant articles were identified by examining title and abstract of the retrieved articles. Third, a final selection was made after reading the full text of the articles. The selection process was conducted by two reviewers independently (I.L-F. and I.A-P), and a third reviewer participated if disagreement (S.M).

For excluded studies, the reason for exclusion was documented.

### Data extraction and synthesis

Following the JBI Manual for Evidence Synthesis [16], we developed a summary sheet to extract the main characteristics and results of the included studies. Data were organised and reported in a table based on major conceptual categories such as: description of general bibliographic data (author(s), year of publication, study country), design of the study, population (sample size and type of cardiovascular event), type of secondary prevention intervention, and main findings related to gender inequalities. Type of intervention was divided into pharmacological treatment, considering both prescription and medication adherence, guideline recommended care, including achievement of the target parameters recommended (guideline goals) and healthy lifestyle recommendations, CR, and use of health services. Studies were reviewed one at a time, combining the studies referred to the same category. We performed a narrative synthesis to accompany the tabulated results and described them more precisely.

## Results

**Fig. 2:**
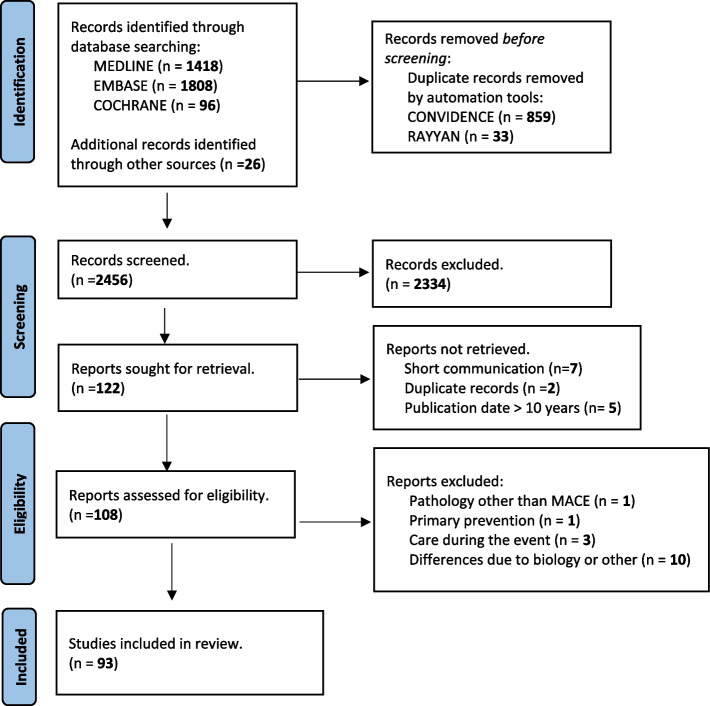
Search Process in PRISMA-ScR Flowchart

As it can be observed in PRISMA flow diagram (Fig. 2), the search strategy produced 3,322 potentially relevant studies. 26 articles were identified manually or by snowball search. After removing 892 duplicates of those remaining, 2,334 were excluded after reviewing the title and abstract and another 14 were not retrieved for their inadequacy according to the eligibility criteria previously defined. After reviewing the full text of the remaining articles [20], another 15 were excluded. The reasons for exclusion were due to studies exploring pathologies other than MACE (*n* = 1), primary prevention (*n* = 1), care during the event (*n* = 3) or differences due to biology or other (*n* = 10). Finally, a total of 93 articles were selected.

In Supplementary material: Additional file 2 to 5, Appendix Tables 1 to 4 present detailed information of the 93 studies included in our scoping review grouped by type of intervention [5, 12, 13, 20–109].

Among the 93 included articles, 27 studies were conducted in European countries, 25 in the USA, 8 in Canada, 7 in Australia and the rest in different world locations. The most common study design was the prospective cohort (*n* = 50, 54%). The pathologies analysed focused on MACE in their various forms, such as acute myocardial infarction, acute coronary syndrome, and stroke.

The following major categories of secondary CVD prevention are described in Table 1. As it can be observed, the main intervention studied in the literature is the pharmacological treatment received after MACE (*n* = 61, 66%), distantly followed by guideline-recommended care (*n* = 26, 28%) and referral to CR (*n* = 16). Finally, there were only 9 publications about gender inequalities in the use of health services after a MACE.

**Table 1:** Articles on the four main secondary prevention interventions for the control of CVD

	**N (%)**	**References**
**Health services use**	**9** (9,68%)	[21, 45, 48, 5, 74, 76, 80, 87, 100]
**Cardiac rehabilitation**	**16** (17.20%)	[22, 34, 41, 44, 52, 5, 56, 58, 77, 84–86, 89, 93, 96, 101]
**Guideline-recommended care**	**26** (27.96%)	
• Healthy lifestyle	9 (9.68%)	[32, 37, 64, 69, 70, 78, 81, 90, 91]
• Guideline goals	21 (22.58%)	[20, 27, 37, 43, 46, 49, 50, 59, 62, 66, 72, 73, 78, 90, 91, 93, 98, 99, 102, 105, 108]
**Pharmacological treatment**	**61** (65.59%)	
• Prescription	56 (60.22%)	[20, 21, 23–25, 28–31, 33, 35, 36, 38, 39, 13, 42, 45–47, 51, 5, 53–55, 57, 58, 60, 62, 63, 12, 65, 66, 68, 72, 74, 75, 78–80, 82, 83, 88, 91, 92, 94, 95, 97, 99–101, 103–107, 109]
• Medication adherence	8 (8.60%)	[26, 40, 61, 67, 71, 74, 91, 101]

*N* Number; % percentage

**Fig. 3:**
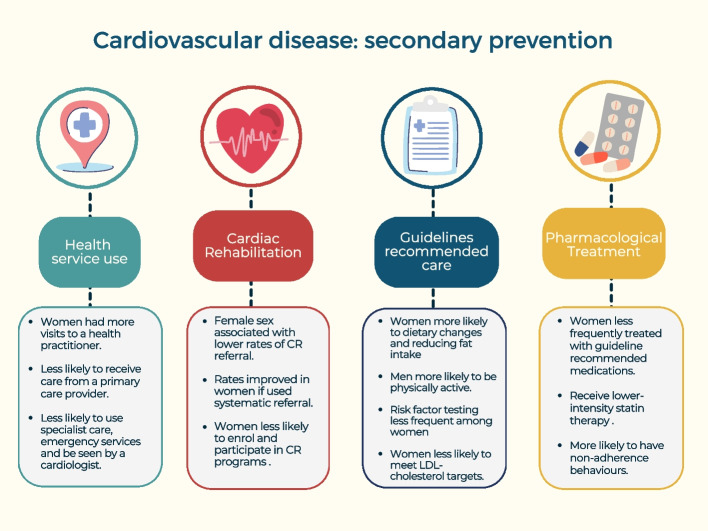
Summary figure of the main findings in the literature

Now we will proceed to explain the main outcomes obtained for each of the secondary prevention interventions. A resume of the main results is shown in Fig. 3.

### Health service use

Understood as the process of accessing and using health services, we found nine studies about health service use. We considered various medical services, such as visits to health practitioners (general practitioners, nurses) or referrals to specialists (cardiologists), number of medical appointments, access to the emergency department, and other forms of health care intervention.

According to medical appointments, some studies showed that the average number of visits to a health practitioner was lower in men, compared to women [45, 48, 5, 100]. However, women were less likely to receive care from a primary care provider, such as, receive dietary advice or have all cardiovascular risk factors measured (low-density lipoprotein-cholesterol or LDL-C, high-density lipoprotein-cholesterol or HDL-C, BP, and haemoglobin A1c or HbA1C) [5, 80, 100]. Women seemed to have worse communication with their healthcare provider and were more likely to receive home health care [80].

In terms of referral to other specialists, two studies found that a greater proportion of men than women used specialist care (79.0% vs. 70.6%, *p* < 0.001), being women less likely to be seen by a cardiologist (77.1% versus 85.7%; *p* < 0.001), and men having almost two more visits on average than women did [45, 74]. On the other hand, the study of Abrahamyan et al. observed similar referral to other specialists across genders, although it was also noted that the majority of patients seen in heart failure clinics were men [21].

Finally, one study showed that men had a greater utilisation of emergency care services (57.6% vs. 52.7%, *p* < 0.001), and more visits to the emergency room (1.32 vs. 1.15 visits, *p* < 0.001) than women [45], but some other agreed that it was women who were more likely to use emergency department services [76, 87].

### Cardiac rehabilitation

CR are a comprehensive, multidisciplinary chronic disease management program that incorporates patient assessment, nutritional counselling, cardiovascular risk factor management, psychological management, exercise training, and physical activity counselling. As a result, substantial evidence indicates that CR participation is effective in improving CVD management [93].

We included sixteen studies on gender differences in referral (referral order from the health practitioner to an outpatient CR program), enrolment (initiating the program), and attendance (participation in most or all of the sessions of the program) of CR programmes, as well as literature on adherence, potential barriers, and reasons for CR dropout.

Four studies found that female sex was associated with lower rates of CR referral [34, 44, 56, 93], while two other papers found no statistically significant gender difference [58, 101]. Gravely et al. [44] studied potential effects of referral strategies on access to CR. This study showed that systematic referral alone resulted in 6.5 times more referrals than usual referral and combined with a liaison facilitated strategy resulted in a ten times greater referral rate. They found that men were significantly more likely to be referred to CR than women using a liaison-facilitated strategy (63.5% and 48.6%, *p* < 0.01), although there were no significant gender differences following systematic referral (70.5% and 68.6%, *p* = 0.70) or the combined approach (85.6% and 84.2%, *p* = 0.73).

Regarding enrolment and attendance, several studies showed that women were less likely to enrol in CR programs and had lower rates of attendance [34, 41, 44, 56]. The odds of women attending a CR program were lower than men being between 0.64–0.87 (95% Confidence interval (95%CI)) [5, 77]. This is similar to the study published by Resurrección et al. [84], which describes that female patients have up to four times higher odds of non-participation (Odds Ratio (OR) range 1.64–4.17).

Another study [93] summarised that even referral rates had increased from 2007, improvements in participation have not been reported, remaining female CR attendance unchanged from 1982. As seen previously [44] and according to the study by Oosenbrug et al. [77], CR enrolment favoured women in studies where patients were systematically referred.

On the other hand, only one study [52] conducted in Korea, found no differences in terms of participation between gender. In terms of adherence to CR programmes, Samayoa et al. [89] concluded that there were no significant gender differences in programs of < 12 weeks; however, in programs of ≥ 12 weeks, men were significantly more adherent than women.

Four studies explored potential barriers and reasons for non-participation and dropout from a CR program in women [56, 85, 86, 96].

The reasons were grouped into similar categories, such as, intrapersonal, interpersonal, logistical barriers, characteristics about the program or health system issues. They are described in detail in supplementary material: Additional file 3: Table 2. The most common reasons were family responsibilities, transport or distance issues, fear of exercise, uncertainty about the purpose of CR, lack of insurance, lack of endorsement and recommendation to attend to the program, and health beliefs about CVD.

Finally, the study by Angus et al. [22] highlights the complexity of adopting new health practices learned in a CR program, showing how people struggled to find a place for health practices in everyday life. So, some participants tried to conceal health-related activities at work to avoid unemployment or tried to minimise family concern by hiding their illness so that they did not seem "weak". They described the importance of the cooperation of others to support lifestyle changes. Participants tried to educate and inspire family members, but they often met with resistance, making it hard to maintain healthy habits.

### Guideline recommended care: healthy lifestyle

Worldwide guidelines recommend adopting healthy lifestyles to prevent CVD. We found several studies that showed no gender differences with regard to get healthy lifestyle advice or education [37, 69, 81, 90, 91]. One study showed that despite small improvements, health behaviours (physical activity, healthy diet, or smoking) remained worse than in general population one year after the event [64].

Women were more likely to report more often dietary changes, such as reducing their dietary fat intake (*p* < 0.001) [37, 69, 90, 91], whereas men seemed to be more physically active [32, 69, 70, 78, 90]. Female gender was an independent predictor of physical inactivity, with women having 1.37 times the odds of being less active from before to 12-months after acute myocardial infarction (95%CI: 1.21–1.55) [70, 78]. Regarding smoking, two studies showed that the rate of persistent smoking/relapse or reduction was similar in both sexes [64, 81].

### Guideline recommended care: guideline goals

Guidelines recommend certain controls and goal targets after a MACE. One study [108] showed that cardiovascular risk factor control was generally suboptimal in both men and women for all risk factors examined (total cholesterol, LDL-C, HDL-C, BP, and glucose targets). On the other hand, several studies [62, 73, 102] agreed that risk factor testing was consistently less frequent among women (*p* < 0.001). Lee et al. [62] also added that those patients who had risk factors assessed more than once increased their odds of target achievement by > 5%.

Regarding gender differences in risk factors measured, less female had their weight measured [37], and significant gender differences were found in BP control and lipid profiles [37, 46]. Naicker et al. [72] showed that women were less likely to have their lipid profiles taken (OR = 1.17 [95%CI, 1.03–1.33]), but were more likely to have two BP measurements (OR = 0.83 [95%CI, 0.71–0.96]), and to be referred to a dietician or weight loss program (OR = 0.87 [95%CI, 0.76–0.98]).

Several studies examined gender differences in the achievement of recommended therapeutic goals. Two of them found no statistically significant differences when considering therapeutic goals, such as, LDL-C, non-HDL-C, BP, HbA1, BMI or physical activity [90, 98].

Regarding control of lipid targets, four studies observed no gender differences [43, 59, 78, 90], while five found that females were less likely to meet the target lipid goal recommended [49, 66, 93, 99, 108]. The number of studies showing gender differences increased when only LDL cholesterol target was considered. Only one study showed no statistically significant gender difference in the achievement of the therapeutic goal (LDL-C < 70mg/dl) [91], compared with seven studies in which women were less likely to achieve LDL-cholesterol targets [20, 27, 37, 49, 62, 102, 105].

Finally, when other therapeutic goals were considered, three studies found that women were less likely to achieve BP targets [46, 50, 59], while two others showed no difference [37, 102]. For HbA1c, one study found a higher proportion of women achieving HbA1c target [62] whereas another showed otherwise [37].

### Pharmacological treatment: prescription

Four main drugs are recommended for secondary prevention: aspirin or other antiplatelet drugs, statins, Beta blockers (BBs), and angiotensin-converting enzyme inhibitors (ACE-I) or angiotensin receptor blockers (ARBs). We found fifty-six studies that examined gender differences in pharmacological treatment. The main results are summarised in Table 2.

**Table 2:** Articles about gender differences in secondary prevention medication in CVD

*Drug*	*Prescription*	*References*	*n*
*Beta-Blockers*	*No gender differences*	[21, 30, 31, 42, 58, 80, 82, 101, 109]	9
	*Women less frequently treated*	[23–25, 28, 31, 35, 36, 38, 5, 12, 13, 39, 45–47, 51, 53, 54, 57, 62, 63, 75, 79, 83, 88, 95, 97, 99, 104, 105, 107]	31
	*Women more frequently treated*	[65, 68, 94]	3
*ACEi/ARB*	*No gender differences*	[21, 57, 58, 82, 95, 109]	6
	*Women less frequently treated*	[29, 31, 35, 36, 38, 39, 13], [45]*, [46]*, [47, 51, 5, 53–55, 62, 63]*, [12, 68]*, [75, 78, 83, 97, 99, 101, 104, 107]	27
	*Women more frequently treated*	[45]**, [46]**, [63]**, [68]**	4
*Statins/ Lipid Lowering*	*No gender differences*	[60, 82, 109]	3
	*Women less frequently treated*	[5, 12, 13, 20, 23–25, 28, 29, 31, 35, 36, 38, 39, 42, 46, 47, 51, 53–55, 57, 58, 62, 63, 66, 68, 72, 74, 78, 80, 83, 88, 91, 95, 97, 99–101, 104–107]	43
	*Women more frequently treated*	[65]	1
*Aspirin/ Antiplatelet*	*No gender differences*	[30, 35, 42, 57, 78, 82, 101]	7
	*Women less frequently treated*	[24, 31, 36, 38, 39, 13, 46, 47, 5, 53, 54, 58, 60, 62, 63, 12, 65, 72, 79, 83, 88, 92, 95, 97, 99, 103, 105, 107, 109]	29
	*Women more frequently treated*		
*Other Antiplatelets*	*No gender differences*	[57, 82, 101]	3
	*Women less frequently treated*	[23, 35, 5, 12, 39, 46, 54, 55, 58, 65, 83, 97, 103, 109]	14
	*Women more frequently treated*		

*n* number, *Only ACEi (Angiotensin converting enzyme ACE inhibitors); **Only ARBs (angiotensin receptor blockers)

As it can be observed, the majority of papers reviewed found that women were less frequently treated than men for all the drugs analysed.

These results were also observed when other comedications frequently used in secondary CVD prevention were analysed (Table 3).

**Table 3:** Articles about gender differences in other secondary prevention medication in CVD

*Drug*	*References*	*n*
*Calcium blocker- channels*	*Women ****more**** frequently treated:* [47, 63, 68, 95, 101]	5
*Anticoagulants*	*Women ****more**** frequently treated:* [47, 95] *Women ****less**** frequently treated:* [42, 45, 53, 101] *No gender differences:* [54]	2 4 1
*Nitrates*	*Women ****more**** frequently treated:* [24, 33, 82, 83] *Women ****less*** frequently treated: [47, 95]	4 2
*Antidiabetics*	*Women ****more**** frequently treated:* [63, 101]	2
*Diuretics*	*Women ****more**** frequently treated:* [63, 68, 88, 95, 101, 107] *Women ****less**** frequently treated: * [75] *No gender differences:* [31]	6 1 1

*N* Number

Also, some studies explored gender differences in adequate statin dose. Twelve studies found that women were less likely than men to receive high-intensity statin therapy (*p* < 0.05) [20, 24, 38, 39, 55, 66, 74, 79, 80, 91, 99, 100].

### Pharmacological treatment: adherence

Medication adherence is defined by the World Health Organization as “the degree to which the person’s behaviour corresponds to the agreed recommendations from a health care provider” [110]. Our search found eight articles that explored adherence to treatment. Four of them showed no statistically significant gender differences [67, 71, 91, 101]. In addition, Setny et al. [91] added that there was no difference in the reasons for treatment discontinuation. On the contrary, the other four found gender differences [26, 40, 61, 74]. Bhuyan et al. [26] observed that cost-related medication non-adherence behaviours were more likely (1.54 times 95%CI, 1.33–1.77) among women, such as skipping medication, taking less medication, or delaying refills.

## Discussion

The most important contribution of this study is to summarize the existing literature on gender inequalities in secondary prevention of CVD. In doing so, we are able to show that gender differences still exist and to emphasise the importance of incorporating a gender perspective in secondary prevention interventions for CVD.

Overall, 93 articles reporting the four main outcomes for secondary prevention of MACE were identified in this scoping review, namely: health service use, CR, guideline recommended care and pharmacological treatment. To begin with, the literature reviewed show the existence of gender differences in terms of health care and management of secondary prevention of CVD. Thus, in relation with the use of health services, women are more likely to engage with primary healthcare [45, 48], probably due to a higher awareness of disease prevention. On the contrary, they were less likely to use specialist care, such as emergency services or to be seen by a cardiologist. The main findings for CR followed the same line; female sex was associated with lower rates of CR referral, although these rates improved when systematic referral was used. So, women are less likely to enroll, participate and adhere to the CR program. When analyzing guideline-recommended care, we found several studies that reported that women were more likely to make dietary changes, such as reducing fat intake. On the other hand, men were more likely to increase their physical activity. Women had lower rates of risk factor testing and they seem to be less likely to reach cholesterol goal targets. Regarding pharmacological treatment, studies showed that women were less frequently treated with guideline-recommended medications, including lower-intensity statins, and they seem more likely to be non-adherent.

According to the use of health services, women are subject to less therapeutic efforts, their cardiovascular risk factors (CVRFs) are less frequently monitored [72, 80, 102, 108] and they are less likely to receive specialist care or to have access to specific treatments, such as CR programs. It could be explained by an androcentric bias in health care (lower number of visits to the emergency room [87], lower cardiology referrals [74]) that has not been internalized or assumed by health professionals or by the general population. The study by Venditti et al. [111] suggests that undertreatment in women might be related to lower awareness of CVD, less social support, low socioeconomic status and lack of insurance, barriers that are thought to limit women's access to care, making it difficult to monitor and control risk factors, disproportionately affecting women and widening gender inequalities. Other studies [21, 45] defend the idea that women receiving less care could be explained by men presenting more exacerbations and because of their clinical characteristics of their comorbidities (e.g. ischemic etiology, reduced left ventricular ejection fraction, age…), although multimorbidity burden has shown to be similar in both sexes. Moreover, the study of Okunrintemi et al. [76], with atherosclerotic cardiovascular disease patients, showed that women reported poor communication with their healthcare providers and poor satisfaction with their healthcare experience, reflecting a potential gender bias among the health providers. Of note, other studies suggest that outcomes may be better for female patients treated by female physicians [112, 113].

Regarding CR programs, women are referred less, consequently, they enroll less, and they also have lower attainment and consecution of these programs. The study by Supervia et al. [96] examined the barriers for women in this area. They identified a complex range of barriers (socioeconomic, demographic, medical and societal), some of them which can be addressed to reduce gender inequalities. Women frequently carry a burden of family responsibilities assuming the family care giver role [34, 56, 85, 93, 96], that is not shared by their male counterparts. This gender roles and social expectations might influence perceptions of health and prioritization of medical care, making women feel compelled to prioritize the needs of others over their own health. Lack of social support, disease awareness, work conflicts, personal health beliefs or perception of exercise as tiring or painful, might take part as well in the lower adherence to the programs.

In line with the guideline recommended care, this gender bias in the use of CR programs is consistent with the integration of other health measures recommended in the guidelines into daily life, such as, dietary changes (reducing fat intake) or engaging with physical activity. The study by Vynckier et al. [114] provides an overview on lifestyle advice given by health professionals and patients' attempts to implement it. When considering in particular actions to adopt a healthier diet, it appears that women are more likely to adopt changes such as salt and calorie reduction but are less likely to be advised to lose weight by participating in physical activity. In addition, it has been widely reported that women are less likely to comply with physical activity recommendations and are less likely to be considered active [69, 70, 78, 90]. Gender stereotypes may lead to women having fewer opportunities or incentives to participate in certain types of physical activity. In fact, being a woman is considered an independent risk factor for physical inactivity [115]. Women face additional workloads and family responsibilities, which limits the time to engage in exercise. Therefore, family and work demands may affect women's ability to incorporate physical activity into their daily routine. Also, the prioritization of other activities (caring for family members) may cause physical exercise to be relegated to the background [114]. Thus, while there is a concern to change eating habits, this is not enough if it is not accompanied by an increase in physical exercise and appropriate health care.

The differences in terms of pharmacological treatment are well documented, from women receiving more obsolete and less current treatment BBs, antiplatelet, second antiplatelet agent, anticoagulation, and statins…) instead of guideline-based medical therapy, to less intensive treatment and lower dosage. The reasons of this inequity, however, remained uncertain [35, 68, 105]. Another study by Vynckier [100] suggests that reduced awareness and underestimation of CVD risk in women might lead to underdiagnosis and undertreatment. The lack of evidence-based guidelines for women and the historical predominance of male participants and women underrepresentation in clinical trials would exacerbate these gender differences. This lack of scientific evidence for women may influence treatment decisions, preventing interventions and treatments from being equally effective for women and men. Barrett et al. added the perception of physicians and patients that women are at lower risk of severe CVD outcomes than men [25]. Furthermore, women are proven to be less adherent to therapeutical treatment than men. Reasons for this might be related to socioeconomic and cultural factors or barriers in access to health care as discussed before [74], that might influence patient adherence as well as prescription decisions in health care professionals. Lack of insurance has been reported as a risk of medication non-adherence [26, 40, 74]. However, prescription by a cardiologist or at hospital discharge has been shown to be a predictor of adherence [25]. This is especially important for statin treatment, as women often express concerns about side effects and a preference for diet or natural remedies and tend to abandon or refuse treatment [74].

Although this scoping review used a rigorous and thorough search strategy, limitations exist. Only peer reviewed articles written in English and Spanish were included in the review. This may have prevented the location of other relevant articles. Our selected search timeline of 2014–2023 could have eliminated other pertinent articles from being identified. However, to discuss gender inequities according to guideline recommendations and potential solutions to reduce them, we consider important to gain insight on the most recent secondary prevention interventions shown in the literature. Another possible limitation could be a publication bias in the literature, whereby studies that shown gender inequities are more likely to be published than those that find no differences.

Every effort was made to identify the various terms used to discuss gender equity and secondary prevention of CVD interventions in the literature to locate relevant articles, both in thesaurus vocabulary and free terms. However, it is possible that some relevant articles that did not include these common terms were excluded. Finally, this scoping review did not provide quality assessment of included studies. Indeed, due to the wide variety of studies found, this review aims to summarise current knowledge, identify gaps, discuss, and find potential measures to reduce gender inequity in secondary prevention of CVD, rather than to assess the quality of the included studies.

## Conclusions

Performing this scoping review allowed us to compile and summarise the existing knowledge regarding the presence of gender inequalities on the secondary prevention of CVD. It also allowed us to propose ways to focus secondary prevention interventions to mitigate these inequalities. Additional research is required to delve into various factors influencing therapeutic disparities, non-participation, or dropout from CR programs, among other aspects. However, our findings underscore the importance of incorporating a gender perspective into evidence-based interventions for CVD secondary prevention. Thus, it is important to develop different strategies to specifically address gender inequalities in health care and the different needs, fears or preferences that men and women might have after a cardiovascular event to reach, assess and treat them in the better way. This will improve existing knowledge about the presentation and treatment of CVD in men and women. This approach is crucial to ensure the most equitable and effective attention to this issue.

### Supplementary Information

Additional File 1.  Search Strategy.

Additional File 2.  Table 1. Summary of publications and main results of Health service use.

Additional File 3.  Table 2. Summary of publications and main results of Cardiac Rehabilitation.

Additional File 4.  Table 3a and b. Summary of publications and main results of Guideline Recommendations.

Additional File 5.  Table 4a and b. Summary of publications and main results of Pharmacological treatment.

## Data Availability

No datasets were generated or analysed during the current study.

## Abbreviations

CVD: Cardiovascular disease
BP: Blood Pressure
CR: Cardiac Rehabilitation
BMI: Body mass index
MACE: Major cardiovascular event
LDL-C: (Low-density lipoprotein-cholesterol
HDL-C: High-density lipoprotein-cholesterol
HbA1C: Haemoglobin A1c
BBs: Beta blockers
ACE-I: Angiotensin-converting enzyme inhibitors
ARBs: Or angiotensin receptor blockers
CVRFs: Cardiovascular risk factors
