# Supplementary material for: Gender inequalities in secondary prevention of cardiovascular disease: a scoping review
Source: Int J Equity Health. 2024 Jul 23;23:146. doi: 10.1186/s12939-024-02230-3 (PMC11264402; doi:10.1186/s12939-024-02230-3)
Supplement: Supplementary file 1 — Additional File 1. Search Strategy. [file 12939_2024_2230_MOESM1_ESM.pdf]

ADDITIONAL FILE 1. SEARCH STRATEGY

**PUBMED MEDLINE**

** Search by MeSH terms and free terms in title and abstract.*

(("cardiovascular diseases"[MeSH Terms] OR "cardiovascular disease"[Title/Abstract] OR (("cardiovascular system"[MeSH Terms] OR "cardiovascular system"[Title/Abstract]) AND ("outcome"[Title/Abstract] OR "outcomes"[Title/Abstract])) OR ("myocardial ischaemia"[Title/Abstract] OR "myocardial ischemia"[MeSH Terms] OR "myocardial ischemia"[Title/Abstract] OR "coronary artery disease"[MeSH Terms] OR "coronary artery disease"[Title/Abstract]) OR ("acute coronary syndrome"[MeSH Terms] OR "acute coronary syndrome"[All Fields]) OR ("acute"[Title/Abstract] AND ("myocardial infarction"[MeSH Terms] OR "myocardial infarction"[Title/Abstract])) OR ("coronary disease"[MeSH Terms] OR "coronary disease"[Title/Abstract] OR "coronary heart disease"[Title/Abstract]) OR ("major"[Title/Abstract] AND ("cardiovascular system"[MeSH Terms] OR "cardiovascular"[Title/Abstract]) AND ("event"[Title/Abstract] OR "events"[Title/Abstract])) OR (("major"[Title/Abstract] AND (("cardiovascular diseases"[MeSH Terms] OR "cardiovascular"[Title/Abstract] OR "cardiac"[Title/Abstract]) AND "adverse"[Title/Abstract] AND "event"[Title/Abstract])) OR "major adverse cardiac event"[Title/Abstract]) OR ("stroke"[MeSH Terms] OR "stroke"[Title/Abstract] OR "acute cerebrovascular accident"[Title/Abstract]) OR ("hemorrhagic stroke"[MeSH Terms] OR "hemorrhagic stroke"[Title/Abstract] OR "haemorrhagic stroke"[Title/Abstract]) OR ("ischemic stroke"[MeSH Terms] OR "ischemic stroke"[Title/Abstract]) OR ("heart failure"[MeSH Terms] OR "heart failure"[Title/Abstract]))

AND

("secondary prevention"[MeSH Terms] OR "secondary prevention"[Title/Abstract] OR "relapse prevention"[Title/Abstract] OR ("health"[Title/Abstract] AND ("check"[Title/Abstract] OR "checked"[Title/Abstract] OR "checkings"[Title/Abstract] OR "checks"[Title/Abstract])) OR ("delivery of health care"[MeSH Terms] OR "delivery of health care"[Title/Abstract] OR "health care delivery"[Title/Abstract]) OR ("patient acceptance of health care"[MeSH Terms] OR "patient acceptance of health care"[Title/Abstract] OR "health care utilization"[Title/Abstract]) OR ("therapy"[MeSH Subheading] OR "therapy"[Title/Abstract] OR "disease management"[Title/Abstract] OR "disease management"[MeSH Terms]) OR ("drug therapy"[MeSH Subheading] OR "drug therapy"[Title/Abstract] OR "drug therapy"[MeSH Terms]) OR ("drug prescriptions"[MeSH

Terms] OR "drug prescriptions"[Title/Abstract] OR "drug prescription"[Title/Abstract]) OR ("medication adherence"[MeSH Terms] OR "medication adherence"[Title/Abstract]) OR (("clinical"[Title/Abstract] AND ("outcome"[Title/Abstract] OR "outcomes"[Title/Abstract])) OR "clinical outcomes"[Title/Abstract]) OR ("patient care"[MeSH Terms] OR "patient care"[Title/Abstract]) OR ("aftercare"[MeSH Terms] OR "aftercare"[Title/Abstract] OR "follow up care"[Title/Abstract]) OR ("rehabilitation"[MeSH Terms] OR "rehabilitation"[Title/Abstract] OR "rehabilitation"[MeSH Subheading]) OR ("cardiac rehabilitation"[MeSH Terms] OR "cardiac rehabilitation"[Title/Abstract]) OR ((("pharmaceutical preparations"[MeSH Terms] OR "pharmaceutical preparations"[Title/Abstract] OR "medication"[Title/Abstract] OR "medications"[Title/Abstract]) AND "use"[Title/Abstract]) OR "medication use"[Title/Abstract]) OR ("prevention and control"[MeSH Subheading] OR "prevention and control"[Title/Abstract]) OR ("recurrance"[Title/Abstract] OR "recurrence"[MeSH Terms] OR "recurrence"[Title/Abstract] OR "recurrences"[Title/Abstract]
OR "recurrencies"[Title/Abstract] OR "recurrency"[Title/Abstract]) OR ("patient readmission"[MeSH Terms] OR "patient readmission"[Title/Abstract]) OR ("referral and consultation"[MeSH Terms] OR "referral and consultation"[Title/Abstract] OR "consultation and referral"[Title/Abstract]))

AND

("gender equity"[MeSH Terms] OR "gender equity"[Title/Abstract] OR
"gender differences"[Title/Abstract] OR "sex differences"[Title/Abstract]))

AND (FILTER)

((y_10[Filter]) AND (fha[Filter]) AND (fha[Filter]) AND (english[Filter] OR spanish[Filter]) AND (alladult[Filter]))

EMBASE

** Thesaurus search: entree term and free terms in title and abstract adapted assisted by the* *Polyglot Search Translator.*

('cardiovascular disease'/exp OR 'cardiovascular disease':ti,ab OR (('cardiovascular system'/exp OR 'cardiovascular system':ti,ab) AND (outcome:ti,ab OR outcomes:ti,ab)) OR ('myocardial ischaemia':ti,ab OR 'heart muscle ischemia'/exp OR 'myocardial ischemia':ti,ab OR 'coronary artery disease'/exp OR 'coronary artery disease':ti,ab) OR 'acute coronary syndrome' OR (acute:ti,ab AND ('heart infarction'/exp OR 'myocardial infarction':ti,ab)) OR ('ischemic heart disease'/exp OR 'coronary disease':ti,ab OR 'coronary heart disease':ti,ab) OR (major:ti,ab AND ('cardiovascular system'/exp OR cardiovascular:ti,ab) AND (event:ti,ab OR events:ti,ab)) OR ((major:ti,ab AND ((' cardiovascular disease'/exp OR cardiovascular:ti,ab OR cardiac:ti,ab) AND adverse:ti,ab AND event:ti,ab)) OR 'major adverse cardiac event'/exp OR 'major adverse cardiac event':ti,ab) OR ('cerebrovascular accident'/exp OR stroke:ti,ab OR 'acute cerebrovascular accident':ti,ab) OR ('hemorrhagic stroke':ti,ab OR 'haemorrhagic stroke':ti,ab) OR ('ischemic stroke'/exp OR 'ischemic stroke':ti,ab) OR ('heart failure'/exp OR 'heart failure':ti,ab))

AND

('secondary prevention'/exp OR 'secondary prevention':ti,ab OR 'relapse prevention':ti,ab OR 'health check'/exp OR 'health check':ti,ab OR 'health care delivery'/exp OR 'delivery of health care':ti,ab OR 'health care delivery':ti,ab OR 'patient attitude'/exp OR 'patient acceptance of health care':ti,ab OR 'health care utilization'/exp OR 'health care utilization':ti,ab OR ('disease management':ti,ab OR 'disease management'/exp) OR ('drug therapy':ti,ab OR 'drug therapy'/exp) OR ('prescription'/exp OR 'drug prescriptions':ti,ab OR 'drug prescription':ti,ab) OR ('medication compliance'/exp OR 'medication adherence':ti,ab) OR ('patient care'/exp OR 'patient care':ti,ab) OR (aftercare/exp OR aftercare:ti,ab OR 'follow up care':ti,ab) OR ('cardiac rehabilitation'/exp OR 'cardiac rehabilitation':ti,ab) OR (((drug/exp OR 'pharmaceutical preparations':ti,ab OR drug:ti,ab OR medication:ti,ab OR medications:ti,ab) AND use:ti,ab) OR 'drug utilization review'/exp OR 'medication use':ti,ab) OR ('prevention and control'/exp OR 'prevention and control':ti,ab) OR (recurrance:ti,ab OR 'recurrence risk'/exp OR recurrence:ti,ab OR recurrences:ti,ab OR recurrencies:ti,ab OR recurrency:ti,ab) O R ('hospital readmission'/exp OR 'patient readmission':ti,ab) OR ('patient referral'/exp OR 'referral and consultation':ti,ab)

AND

('gender equity'/exp OR 'gender equity':ti,ab OR 'gender differences':ti,ab OR 'sex differences':ti,ab))

AND (FILTER)

(2014:py OR 2015:py OR 2016:py OR 2017:py OR 2018:py OR 2019:py OR 2020:py OR 2021:py OR 2022:py OR 2023:py) AND 'human'/de AND ([adult]/lim OR [aged]/lim OR [very elderly]/lim) AND 'article'/it AND ([english]/lim OR [spanish]/lim)

COCHRANE

** Search by MeSH and free terms in title and abstract adapted* assisted by the *Polyglot Search Translator.*

([mh "gender equity"] OR "gender equity":ti,ab OR "gender differences":ti,ab OR "sex differences":ti,ab) AND ([mh "cardiovascular diseases"] OR "cardiovascular disease":ti,ab OR "myocardial ischaemia":ti,ab OR [mh "myocardial ischemia"] OR "myocardial ischemia":ti,ab OR [mh "coronary artery disease"] OR "coronary artery disease":ti,ab OR [mh "acute coronary syndrome"] OR "acute coronary syndrome" OR [mh "myocardial infarction"] OR "myocardial infarction":ti,ab OR "coronary heart disease":ti,ab OR "major adverse cardiac event":ti,ab OR [mh stroke] OR stroke:ti,ab OR "acute cerebrovascular accident":ti,ab OR [mh "hemorrhagic stroke"] OR "hemorrhagic stroke":ti,ab OR "haemorrhagic stroke":ti,ab OR [mh "ischemic stroke"] OR "ischemic stroke":ti,ab OR [mh "heart failure"] OR "heart failure":ti,ab) AND ([mh "secondary prevention"] OR "secondary prevention":ti,ab OR "relapse prevention":ti,ab OR "health check":ti,ab OR [mh "delivery of health care"] OR "delivery of health care":ti,ab OR "health care delivery":ti,ab OR [mh "patient acceptance of health care"] OR "patient acceptance of health care":ti,ab OR "health care utilization":ti,ab OR "disease management":ti,ab OR [mh "disease management"] OR [mh "drug therapy"] OR "drug therapy":ti,ab OR [mh "drug prescriptions"] OR "drug prescriptions":ti,ab OR "drug prescription":ti,ab OR [mh "medication adherence"] OR "medication adherence":ti,ab OR "clinical outcomes":ti,ab OR [mh "patient care"] OR "patient care":ti,ab OR [mh aftercare] OR aftercare:ti,ab OR "follow up care":ti,ab OR [mh "cardiac rehabilitation"] OR "cardiac rehabilitation":ti,ab OR "medication use":ti,ab OR [mh "prevention and control"] OR "prevention and control":ti,ab OR recurrance:ti,ab OR [mh recurrence] OR recurrence:ti,ab OR [mh "patient readmission"] OR "patient readmission":ti,ab OR [mh "referral and consultation"] OR "referral and consultation":ti,ab)
