# Supplementary material for: Gender inequalities in secondary prevention of cardiovascular disease: a scoping review
Source: Int J Equity Health. 2024 Jul 23;23:146. doi: 10.1186/s12939-024-02230-3 (PMC11264402; doi:10.1186/s12939-024-02230-3)
Supplement: Supplementary file 2 — Additional File 2. Table 1. Summary of publications and main results of Health service use. [file 12939_2024_2230_MOESM2_ESM.pdf]

ADDITIONAL FILE 2

Table 1. Summary of publications and main results of **Health service use**

Ref	Author	Year	Country	Study Design	Sample characteristics	Main findings
5	Hyun K. et al.	2021	Australia	Prospective cohort	n= 9283 subjects with Acute coronary [illegible]	Women were more likely to visit a general practitioner (p<0001) but less likely to receive dietary advice (p<0.05) [illegible]
21	Abrahamyan, L. et al.	2018	Canada	Cross-sectional	n= 884 subjects with Heart Failure	More men than women are treated in specialized HF clinics, but similar care is provided to both genders.
45	Gutiérrez, A.G. et al.	2020	Spain	Cross-sectional	n= 17516 subjects with Heart failure	More women visited general practitioner after HF, although annual visits were similar in both sexes. Women visited nurses more. Greater proportion of men used specialist care and had almost two more visits than women. Men showed greater utilization of emergency care and more visits to the emergency room.
48	Höhn, A et al.	2020	Denmark	Prospective cohort	n= 24146 subjects with Stroke; and<br>n= 17215 subjects with Myocardial infarction	Men had lower levels of primary health-care use than women, before and after hospitalization.<br>After hospitalization those differences were more pronounced.
74	Nanna MG. et al.	2019	US	Cross-sectional	n = 5693 subjects with Guideline indication for statin treatment	Women were less likely to be seen by a cardiologist. (p<0.001).
76	Okunrintemi, V et al.	2018	US	Cross-sectional	n = 21353 subjects with Atherosclerotic Cardiovascular disease	Women had worse communication with healthcare providers, were more likely to use emergency department, and they tend not to follow treatment.
80	Rachamin, Y. et al.	2021	Switzerland	Cross-sectional	n = 2398 subjects with Cardiovascular disease	Men were more likely to have all risk factors measured (LDL-C, BP and HbA1C, whereas women were more likely to have one or none of the risk factors assessed.
87	Roth, D.L. et al.	2016	US	Prospective cohort	n = 279 subjects with Stroke	Women were more likely than men to receive home health care and to use emergency department services during post-acute care.
100	Virani, S.S. et al.	2015	US	Prospective cohort	n= 972532 subjects with Cardiovascular disease	Female patients with CVD had received more primary care visits than male patients but were less likely to receive care from a physician primary care provider (as opposed to a nurse practitioner

HF: Heart Failure; LDL-C: low-density lipoprotein-cholesterol; BP: blood pressure; HbA1C: hemoglobin A1c; CVD: cardiovascular disease.
