# Supplementary material for: Gender inequalities in secondary prevention of cardiovascular disease: a scoping review
Source: Int J Equity Health. 2024 Jul 23;23:146. doi: 10.1186/s12939-024-02230-3 (PMC11264402; doi:10.1186/s12939-024-02230-3)
Supplement: Supplementary file 3 — Additional File 3. Table 2. Summary of publications and main results of Cardiac Rehabilitation. [file 12939_2024_2230_MOESM3_ESM.pdf]

ADDITIONAL FILE 3

**Table 2.** Summary of publications and main results of **Cardiac Rehabilitation**

| Ref | Author | Year | Country | Study Design | Sample characteristics | Main findings |
|---|---|---|---|---|---|---|
| 5 | Hyun Karice. et al. | 2021 | Australia | Prospective cohort | n= 9283 subjects with Acute coronary syndrome | Women were less likely to participate in cardiac rehabilitation (p<0.001). The odds of women attending a cardiac rehabilitation program 6 to 12 months after the event were lower than men. |
| 22 | Angus, J.E. et al. | 2018 | Canada | Qualitative | n = 32 subjects with cardiovascular disease and Diabetes mellitus | CR patients were more concern to restore family or employment issues than to achieve CR goals. They had difficulty finding place for new health practices, trying to conceal them with work or minimizing the concerns with their family. Cooperation of others was related to achieve lifestyle changes. |
| 34 | Colbert, J.D. et al. | 2014 | Canada | Prospective cohort | n = 25958 subjects with Acute coronary syndrome | Barriers for CR participation in women:<br>**Referral**: older age, lack of written referral necessary for participation, lack of CR insurance.<br>**Enrolment**: perception of exercise as tiring or painful, comorbidities, lack of awareness, strong endorsement to attend, health care provider support or social support system (family, friends), transportation and financial issues, family responsibilities, unemployment.<br>**Completion**: comorbidities, obesity, depression, anxiety, transportation, distance, family responsibilities, divorce, lack of insurance. |
| 41 | El Missiri, A.M. et al. | 2020 | Egypt | Prospective cohort | n = 60 subjects with Ischemic Heart Disease | Women were less likely to be enrolled, time to enroll was prolonged than men, and were less likely to achieve benefits of CR.<br>Men were more adherent attending and women more likely to withdraw. |
| 44 | Gravely, S. et al. | 2014 | Canada | Prospective cohort | n= 1809 subjects with coronary artery disease | Female sex was predictor of CR non-referral, like age > 75, smoking condition, or DM, which were also predictor of non-completion. |
| 52 | Hwan Kim, Y. et al | 2019 | Korea | Prospective cohort | n = 144 subjects with Acute Myocardial infarction | No difference was found in terms of **participation** rate between male and female. There was a very low participation rate in the final CR session. |

CR: Cardiac rehabilitation; DM: diabetes mellitus; CI: confidence interval; CVD: cardiovascular disease.

**Table 2.** Summary of publications and main results of **Cardiac Rehabilitation** (Continue)

| Ref | Author | Year | Country | Study Design | Sample characteristics | Main findings |
|---|---|---|---|---|---|---|
| 56 | Khadanga, S. et al. | 2021 | US | Literature review | n = 80 articles about CR in patients with cardiovascular disease | Women have lower rates of referral and are less likely to have CR recommended, participate, adhere, or complete CR.<br>Determinants of non-participation: low educational status, social isolation and obligations, lack of insurance, support and CR awareness, older age, transportation, unemployment, and fear of exercise. |
| 58 | Khraishah, H. et al. | 2021 | India | Clinical trial | n = 21374 subjects with Acute Myocardial infarction | No significant difference between men and women in cardiac rehabilitation referral (28.9% vs 28.2%) |
| 77 | Oosenbrug MAE. Et al. | 2016 | Canada | Meta-analysis | n = 26 studies about Acute coronary syndrome | Rates of CR enrolment among women are significantly lower compared with men. Women are 36% less likely to enroll CR. CR enrolment favored women in studies in which patients were systematically referred to CR. |
| 84 | Resurrección, D.M et al. | 2019 | Multiple | Systematic Review | n= 63425 subjects with cardiovascular disease, acute coronary syndrome myocardial infarction. | Female patients had higher odds of CR non-participation (OR 1.64–4.17).<br>Discrepancy on dropout rates: some studies associated dropout with male gender, others female gender (95% CI 5.59). |
| 85 | Resurrección, D.M. et al. | 2018 | Spain | Qualitative | n = 10 women with cardiovascular disease | **Reasons for CR dropout**:<br>Personal: self-reported bad health, health beliefs about CVD, family caregiver role, work conflicts. Logistical barriers: transport, timing, distance. Characteristics about CR programs: uncertainty about purpose, expecting more exercise and less counselling, needs for equipment.<br>Health system: financial assistance, waiting list, delays on referral |

CR: Cardiac rehabilitation; DM: diabetes mellitus; CI: confidence interval; CVD: cardiovascular disease.

**Table 2.** Summary of publications and main results of **Cardiac Rehabilitation** (Continue 2)

| Ref | Author | Year | Country | Study Design | Sample characteristics | Main findings |
|---|---|---|---|---|---|---|
| 86 | Resurrección, D.M. et al. | 2017 | Multiple | Systematic Review | n= 4617 women with cardiovascular disease | Barriers for women's nonparticipation or drop out from CR are related to gender conceptions such us their health is not important, they have to support their family, or logistic difficulties to get there. |
| 89 | Samayoa L. et al. | 2016 | Canada | Meta-analysis | n = 8176 (14 studies) about subjects with Acute coronary syndrome | CR adherence is significantly lower among women than men, although there were no sex differences in short CR programs (< 12 weeks). |
| 93 | Smith, J.R. et al. | 2022 | US | Literature review | n = 150 articles about CR | Females were less likely to be referred, enroll, attend, and complete CR programs.<br>Referral rate has increased but not participation.<br>Female Barriers: transportation problems, family responsibilities, medical issues, perception of exercise as tiring o painful. |
| 96 | Supervía, M. et al. | 2017 | US | Systematic Review | n = 54 studies about subjects with Myocardial infarction, heart failure, angina | Women barriers included lower education, lack of social support, and burden of family responsibilities.<br>Automatic referral, assisted enrolment, inceptive-based strategies, and home-based programs, improve CR participation and completion rates. |
| 101 | Vynckier, P. et al. | 2021 | Multiple | Prospective cohort | n = 8261 subjects with Coronary event | No significant gender differences in prescribed (47.7% vs. 42.0%; P = 0.10) and attended (69.5% vs. 67.0%; P = 0.63) CR programs. |

CR: Cardiac rehabilitation; DM: diabetes mellitus; CI: confidence interval; CVD: cardiovascular disease.
