# Supplementary material for: Gender inequalities in secondary prevention of cardiovascular disease: a scoping review
Source: Int J Equity Health. 2024 Jul 23;23:146. doi: 10.1186/s12939-024-02230-3 (PMC11264402; doi:10.1186/s12939-024-02230-3)
Supplement: Supplementary file 4 — Additional File 4. Table 3a and b. Summary of publications and main results of Guideline Recommendations. [file 12939_2024_2230_MOESM4_ESM.pdf]

ADDITIONAL FILE 4

Table 3a. Summary of publications and main results of **Guideline Recommendations: Healthy Lifestyle**

Ref	Author	Year	Country	Study Design	Sample characteristics	Main findings
32	Cewers, E. et al.	2019	Israel	Qualitative	12 healthcare providers interviewed	Health care providers feel that "men and women are equal, but different". They described that they gave different reasons and barriers to being active and that they perform different types of activities.
37	De Smedt, D. et al.	2016	Europe	Prospective cohort	n = 7998 subjects with a Coronary event	Health behaviour of ACS patients was worse than general population. Despite small improvements 1-year post-ACS (increase of fruits and vegetables intake, improvement of dietary habits and physical activity, quit smoking), they remained worse with no gender differences.
64	Leung Yinko, S.S.L. et al.	2015	Multiple	Prospective cohort	n = 740 subjects with Acute coronary syndrome (18-55 years)	No gender differences about dietary, physical activity and weight advice, as well as physical activity and weight changes.<br>More women had tried to eat healthier and quit smoking, although more male had received advice to quit smoking.
69	Maleki, A. et al.	2016	Iran	Cross-sectional	n = 412 subjects with Coronary artery disease	There were no differences between gender and general healthy lifestyle, but there were in subscales.<br>Means of nutrition and avoiding tobacco and alcohol were higher in women. Means of physical activity, health responsibility, and health-based purchases were higher in men.
70	Minges, KE. et al.	2020	Multiple	Prospective cohort	n = 3572 subjects with Acute myocardial infarction	Women were less likely to meet physical activity recommendations and were considered inactive.<br>About 35% change physical activity levels from pre-AMI to 12 months post-AMI.<br>Both men and women increased physical activity one month after AMI and then declined at 12 months.
78	Perera, S. et al.	2021	Australia	Prospective cohort	n = 729 subjects with Coronary artery disease	Healthy lifestyle adherence was 56.7% with no gender difference. Women were less likely to smoke and to be physically active.

ACS: Acute coronary syndrome; AMI: Acute myocardial infarction.

Table 3a. Summary of publications and main results of **Guideline Recommendations: Healthy Lifestyle** (Continue)

Ref	Author	Year	Country	Study Design	Sample characteristics	Main findings
81	Rahman, MA. et al.	2015	Australia	Systematic Review	n = 36591 subjects with Coronary heart disease (16 studies).	The rate of persistent smoking/relapse was high and there was no gender difference.
90	Setny, M. et al.	2022	Poland	Prospective cohort	n = 1025 subjects with Coronary heart disease	There were no gender differences in terms of receiving information about lifestyle changes and introducing them in daily life. Women reported more often a reduction of dietary fat intake, while men declared that they engaged in physical activity.
91	Setny, M. et al.	2021	Poland	Prospective cohort	n = 1236 subjects with Acute coronary syndrome	Both genders were equally educated about hypercholesterolemia management, more frequently by a cardiologist or a family doctor.<br>Women reported more that they reduced their dietary fat intake but there were no gender differences in the frequency of lifestyle changes.

ACS: Acute coronary syndrome; AMI: Acute myocardial infarction.

Table 3b. Summary of publications and main results of **Guideline Recommendations: Guideline Goals**

Ref	Author	Year	Country	Study Design	Sample characteristics	Main findings
20	Yu, B. et al.	2015	China	Clinical trial	n= 9420 subjects with Coronary heart disease	Fewer women attained their LDL-C and non-HDL-C goal. 6.8% of gender disparity was attributable to the gender [illegible]
27	Bird, C.E. et al.	2018	US	Cross-sectional	n = 114918 subjects with Coronary artery disease	Women were more likely to have worse LDL control, OR 1.88 (95% CI, 1.65–2).
37	De Smedt, D. et al.	2016	Europe	Prospective cohort	n = 7998 subjects with Coronary event	No gender difference was seen regarding blood pressure on target. Smaller proportion of females had their weight measured, LDL-C on target, and adequate physical activity.<br>A significant gender by age interaction was seen with blood pressure and cholesterol measured and HbA1c awareness.
43	Gómez-Barrado, J.J. et al.	2017	Spain	Cross-sectional	n = 741 subjects with Coronary artery disease	Approximately half of patients did not achieve their target lipid levels as defined in the European guidelines with no gender differences.
46	Hambraeus, K. et al.	2016	Sweeden	Prospective cohort	n= 39094 subjects with Acute myocardial infarction	Significant gender differences were seen in blood pressure and cholesterol control rate. Slightly more women than men seem to participate in exercise training. ($p<0.001$), but no gender difference was found in smoking cessation rate.
49	Hopstock, L.A. et al	2018	Norway	Prospective cohort	n = 395 (cohort I)<br>n = 132 (cohort II)<br>subjects with Myocardial infarction	Men had a larger decrease in lipid levels compared to women (total cholesterol, and LDL), and had higher odds of treatment target achievement.
50	Hopstock, L.A. et al.	2018	Norway	Prospective cohort	n = 396 (cohort I)<br>n = 131 (cohort II)<br>subjects with Myocardial infarction	Women were less likely to achieve blood pressure target ($p<0.05$) in the first cohort. There was no gender difference in goal achievement in the second cohort.

LDL: low-density lipoprotein; OR: odds ratio; CI: confidence interval; LDL-C: low-density lipoprotein-cholesterol; HbA1C: hemoglobin A1c.

Table 3b. Summary of publications and main results of **Guideline Recommendations: Guideline Goals** (continue)

Ref	Author	Year	Country	Study Design	Sample characteristics	Main findings
59	Koçyiğit, D. et al.	2018	Turkey	Prospective cohort	n = 446 subjects with Coronary artery disease	Obesity, higher blood glucose level and hypertension were more common in females after the event. Smoking was more common in males. Continuation with treatment, increase in physical activity, weight loss or LDL-C, HDL-C and HbA1c levels did not differ between genders.
62	Lee, C.M.Y. et al.	2019	Australia	Cross-sectional	n= 130926 subjects with Coronary Heart disease	Higher proportions of women than men achieved targets for most risk factors (HDL-cholesterol, triglycerides, HbA1c, smoking and BMI).
66	Lu, Y. et al.	2017	US	Prospective cohort	n = 2219 subjects with Myocardial Infarction (18-55 years)	Young women had slightly favourable lipid and lipoprotein profiles compared with men. Women had higher levels of HDL-C but lower levels of TC/HDL-C ratio and LDL. These differences persisted after adjusting ($P<0.05$).
72	Naicker, K. et al.	2014	Canada	Cross-sectional	n= 4931 subjects with Cardiovascular care	Women were significantly less likely than men to have their lipid profiles taken. They were more likely to be referred to a dietician or a weight loss program, to have blood pressure measured, and equally likely to receive referrals for smoking cessation counselling or programs.
73	Nakano A. et al.	2016	Denmark	Prospective cohort	n= 24308 subjects with Heart Failure	Women receive less recommended processes of care than men
78	Perera, S. et al.	2021	Australia	Prospective cohort	n = 729 subjects with Coronary artery disease	Baseline HDL was higher in women, with no gender difference in mean change from baseline to 12 months in TC, LDL-C or triglycerides.
90	Setny, M. et al.	2022	Poland	Prospective cohort	n = 1025 subjects with Coronary Heart disease	For most of the goals treatments, an improvement was observed after the event, although a large percentage did not achieve the target, with no significant gender differences until subdivided. Considering cholesterol levels, more pronounced disproportions were observed in older groups.

LDL-C: low-density lipoprotein-cholesterol; HDL-C: high-density lipoprotein-cholesterol; HbA1C: hemoglobin A1c; BMI: body mass index; TC: total cholesterol.

Table 3b. Summary of publications and main results of **Guideline Recommendations: Guideline Goals** (continue 2)

Ref	Author	Year	Country	Study Design	Sample characteristics	Main findings
91	Setny, M. et al.	2021	Poland	Prospective cohort	n= 1236 subjects with Acute coronary syndrome	There were no significant gender differences in therapeutic goal achievement, although with age, men showed an increased tendency to maintain recommended LDL-C level.
93	Smith, J.R. et al.	2022	US	Literature review	n= 150 studies about subjects with Cardiovascular care	Females are less likely to meet guideline recommended target goals for lipid, diabetes, hypertension, and weight.
98	Turk-Adawi KI. et al.	2016	US	Prospective cohort	n = 8929 subjects with Acute coronary syndrome	Women were less likely to achieve recommended goals after CR for triglycerides, physical activity and HbA1C. However, they were significantly more likely to achieve the HDL goal. There were no gender differences for blood pressure, total cholesterol, LDL, BMI, or smoking cessation.
99	Victor, B.M et al.	2014	US	Prospective cohort	n = 9950 subjects with Coronary artery disease	Women were less likely to achieve LDL and none-HDL cholesterol goal compared with men ($p<0.001$).
102	Vynckier P. et al.	2022	Multiple	Prospective cohort	n = 8261 subjects with Coronary Heart disease	Women had a worse risk factor control compared with men and were less likely to reach recommended levels of physical activity, LDL-C target, to be non-obese and to use lipid-lowering medication.<br>No gender differences were observed in smoking cessation, hypertension treatment and achieving blood pressure target, although women were more likely to have controlled hypertension.
105	Xia, S et al.	2020	China	Cross-sectional	n = 5454 subjects with Cardiovascular disease	Women were more likely to have adequate BP control but less likely to meet recommended LDL-C and physical activity target.
108	Zhao, M. et al.	2020	Multiple	Prospective cohort	n = 10112 subjects with Coronary Heart disease	Overall, risk factor management for the secondary prevention of CHD was generally worse in women than in men. Compared with men, women were less likely to achieve

LDL-C: low-density lipoprotein-cholesterol; CR: cardiac rehabilitation; HbA1C: hemoglobin A1c; HDL: high-density lipoprotein; BMI: body mass index; BP: blood pressure.
