# Supplementary material for: Gender inequalities in secondary prevention of cardiovascular disease: a scoping review
Source: Int J Equity Health. 2024 Jul 23;23:146. doi: 10.1186/s12939-024-02230-3 (PMC11264402; doi:10.1186/s12939-024-02230-3)
Supplement: Supplementary file 5 — Additional File 5. Table 4a and b. Summary of publications and main results of Pharmacological treatment. [file 12939_2024_2230_MOESM5_ESM.pdf]

ADDITIONAL FILE 5

Table 4a. Summary of publications and main results of Pharmacological treatment: Prescription

Ref	Author	Year	Country	Study Design	Sample characteristics	Main findings
21	Abrahamyan, L. et al.	2018	Canada	Cross-sectional	n= 884 subjects with Heart Failure	Men were prescribed higher number of cardiovascular medications, although there were no gender differences in prescription of ACE-I/ARBs and BBs.
23	Arora, S. et al.	2019	US	Cross-sectional	n = 8737 subjects with Myocardial infarction (35-54 years)	Young women had lower probability of receiving lipid-lowering therapies, non-aspirin antiplatelets and BB.
24	Ballo, P. et al.	2016	Italy	Retrospective cohort	n = 2088 subjects with Heart failure	BBs and antiplatelet agents were more prescribed in men, but nitrates were more common in women. Female gender was associated with reduced probability of statin prescription (P<0.0001) and 48% relative increase in the risk of inadequate dose.
25	Barrett, E. et al.	2021	Australia	Prospective cohort	n= 8278 subjects with Cardiovascular disease (MI and stroke)	Women were less likely to use secondary preventive medication and more likely to use neither after the event. A decrease in adherence over time was observed.
28	Birkemeyer, R. et al.	2014	Germany	Prospective cohort	n= 1104 subjects with ST elevation Myocardial infarction	At discharge, prescription of BB and lipid-lowering drugs was significantly lower in women (p < 0.01), persisting at 12 months.
29	Blumer, V. et al.	2021	US	Clinical trial	n= 7141 subjects with Heart Failure	Women were less likely to receive ACE-I/ARB and BB therapy
30	Carcel, C. et al.	2019	US	Meta-analysis	n = 19652 subjects with Stroke	No gender differences in prescription of antihypertensive or antiplatelet agents at 3-month follow-up.
31	Carlson, B. et al.	2020	Mexico	Cross-sectional	n= 155 subjects with Heart failure	At discharge, women were less likely to receive ACE-I/ARB. There were no differences in prescription of BBs, statins, and diuretics.
33	Cho, K.I. et al.	2016	Korea	Prospective cohort	n = 5200 subjects with Acute Myocardial Infarction	The use of antiplatelet drugs, antihypertensive drugs, statins and antiangina drugs except nitrates, at discharge, were lower in women.

ACE-I: Angiotensin-converting enzyme; ARBs: Angiotensin receptor blockers: BBs: beta blockers.

Table 4a. Summary of publications and main results of **Pharmacological treatment: Prescription** (Continue)

Ref	Author	Year	Country	Study Design	Sample characteristics	Main findings
35	Dagan, M. et al.	2022	Australia	Prospective cohort	n = 20976 subjects with Acute coronary syndrome	Women were more likely to be prescribed suboptimal medical therapy (P < 0.001). There was no difference for prescription of aspirin. Women were less likely to be prescribed statins, ACEi/ARB and BB (P< 0.001).
36	Davis, M. et al.	2015	US	Prospective cohort	n= 3237 subjects with Acute coronary syndrome (18-55 years)	Compared to young men, young women were less likely to receive goal-directed medications such as aspirin, beta blockers, lipid-lowering agents, and ACE inhibitors.
38	De Filippis EM. et al.	2020	US	Prospective cohort	n = 2097 subjects with Myocardial infarction	Women were significantly less likely to be discharged on aspirin and BBs (p<0.05), ACEI/ARBs and statins (p<0.001). High-intensity statins were less prescribed in women (p<0.001).
39	Dhruva, S.S. et al.	2022	US	Retrospective cohort	n = 1063973 subjects with Coronary artery disease and Heart Failure	Women had lower odds to receive medication than men. 30 to 90 days after the event women were prescribed less ACE-I/ARB, BBs, aspirin, clopidogrel, and lipid-lowering therapy (p<0.001)
13	Elgendy, I.Y. et al.	2022	US	Prospective cohort	n= 3119 subjects with Myocardial infarction	Women were less likely to be discharged on aspirin, P2Y12 receptor inhibitors, BBs, ACE-I/ARB and statins.
42	Eriksson M. et al.	2021	Sweeden	Prospective cohort	n = 335183 subjects with Stroke	There were no gender differences in prescription of antihypertensives and antiplatelets. Men remained more likely to be prescribed statins, but anticoagulant treatment increased in women ending higher at the end of the study.
45	Gutiérrez, A.G. et al.	2020	Spain	Cross-sectional	n= 17516 subjects with Heart failure	Guideline recommended drugs, such as BBs, ACE-I and aldosterone antagonist, were less frequently dispensed in women, with the exception of ARB.<br>The joint dispensing of the four drugs was more frequent in men, who also received more anti-aggregation and anti-coagulation drugs (p < 0.001).

ACE-I: Angiotensin-converting enzyme; ARBs: Angiotensin receptor blockers: BBs: beta blockers; P2Y12: purinergic receptor P2Y, G-protein coupled, 12 proteins.

Table 4a. Summary of publications and main results of **Pharmacological treatment: Prescription** (Continue 2)

Ref	Author	Year	Country	Study Design	Sample characteristics	Main findings
46	Hambraeus, K. et al.	2016	Sweeden	Prospective cohort	n= 39094 subjects with Acute myocardial infarction	Women used less ACE-I but more ARB than men (p<0.001). Small statistically significant gender difference was shown in the use of ASA, other antiplatelet drugs, statins and BBs.
47	Hassanein M. et al.	2018	Egypt	Prospective cohort	n = 1634 subjects with Acute Heart Failure	ACE-I, BBs, MRA, antiplatelets, statins, and nitrates were less frequently prescribed to women. Digoxin, amiodarone, anticoagulants, and CCB was received more often.
51	Humphries, K.H. et al.	2018	Canada	Prospective cohort	n = 6907 subjects with Major cardiovascular event	Within 90 days of discharge, females were less likely to use ACE-I/ARBs, BB, or statins, irrespective of cardiac troponins level or chest pain type.
5	Hyun K. et al.	2021	Australia	Prospective cohort	n= 9283 subjects with Acute coronary syndrome	6 to 12 months after the event, women were less likely to use indicated secondary prevention medications (p<0.001) than men.
53	Jackson, A.M. et al.	2020	England	Prospective cohort	n = 7878 subjects with Myocardial infarction and angina	In NSTEMI, women were less frequently treated with antiplatelets, statins, BBs and ACE-I/ARBs than men (P<0.05). Patients with STEMI, sex was not an independent predictor of treatment with antiplatelets, statins, ACE-I/ARBs, or BBs.
54	Jortveit, J. et al.	2016	Norway	Cross-sectional	n = 26447 subjects with Myocardial infarction	Fewer women were discharged with secondary prophylactics treatment, particularly statins, with no gender difference in the use of anticoagulants.
55	Kerola, A.M. et al.	2022	Finland	Prospective cohort	n= 8888 subjects with Myocardial infarction (18-54 years)	Women used P2Y12 inhibitors, ACE-I/ARBs, and statins less frequently. Statins dosage was higher among men with corresponding baseline features.
57	Khan, E. et al.	2018	Australia	Prospective cohort	n = 2898 subjects with ST elevation Myocardial infarction	Significantly lower proportion of women received BB and statins. There were no differences for receiving 2nd antiplatelet agents, aspirin, or ACE-I/ARB.
58	Khraishah, H. et al.	2021	India	Clinical trial	n= 21374 subjects with Myocardial infarction	There was statistically significant difference between discharge treatment with aspirin, 2nd antiplatelet and statins, being more prescribed in men.

ACE-I: Angiotensin-converting enzyme; ARBs: Angiotensin receptor blockers; ASA: acetylsalicylic acid; MRA: Mineralocorticoid Receptor Antagonists; CCB: Calcium channel blockers; NSTEMI: Non-ST-elevation myocardial infarction; STEMI: ST-elevation myocardial infarction; P2Y12: purinergic receptor P2Y, G-protein coupled, 12 proteins.

Table 4a. Summary of publications and main results of **Pharmacological treatment: Prescription** (Continue 3)

Ref	Author	Year	Country	Study Design	Sample characteristics	Main findings
60	Ladapo, J.A. et al.	2019	US	Prospective cohort	n = 2213 subjects with Ischemic Heart disease	Women were less likely than men to be taking aspirin at follow-up, but there were no significant differences in statin therapy.
62	Lee, C.M.Y. et al.	2019	Australia	Cross-sectional	n= 130926 subjects with Coronary Heart disease	Younger patients were less likely to be prescribed with the recommended medications (antiplatelet, ACE/ARB, BBs, and statins). Women were even less likely compared with men in the same age group. Younger women age group, were underprescribed.
63	Lee, C.Y. et al.	2021	Malaysia	Prospective cohort	n= 35232 subjects with Acute coronary syndrome	Women were less likely to receive aspirin, statins, BB and ACE-I. Use of ARBs, diuretics, CCB and antidiabetic treatment were significantly higher in women.
12	Leurent, G. et al.	2014	France	Cross-sectional	n= 5000 subjects with ST elevation Myocardial infarction	The rate of prescription of guideline recommended treatments, showed under prescription for women in aspirin, clopidogrel, BB, ACE-I and statins.
65	Lin, C.-F. et al.	2014	China	Prospective cohort	n= 32821 subjects with Acute coronary syndrome	Female patients had lower likelihood of receiving aspirin and clopidogrel, but higher likelihood of receiving BBs and statins than male patients.
67	Lu, Y. et al.	2017	US	Prospective cohort	n = 2219 subjects with Myocardial Infarction (18-55 years)	Compared with men, women had similar use of any statins 1 month after discharge, although they had slightly less use at discharge. Women were less likely to report being treated with a high-intensity dose.
68	Madika, A.L. et al.	2019	France	Prospective cohort	n = 4184 subjects with Coronary artery disease	Women were less likely to receive ACE -I and statins, but more likely to receive ARBs, calcium antagonists, diuretics, and hypertensive medication.
72	Naicker, K. et al.	2014	Canada	Cross-sectional	n= 4931 subjects with Cardiovascular disease	Women were significantly less likely to be prescribed lipid lowering medication and aspirin. Male and female smokers were equally likely to be prescribed smoking cessation medications.

ACE-I: Angiotensin-converting enzyme; ARBs: Angiotensin receptor blockers; BBs: beta blockers; CCB: Calcium channel blockers.

Table 4a. Summary of publications and main results of Pharmacological treatment: Prescription (Continue 4)

Ref	Author	Year	Country	Study Design	Sample characteristics	Main findings
74	Nanna MG. et al.	2019	US	Cross-sectional	n = 5693 subjects with Guideline indication for statin treatment	Women were significantly less likely to be on a statin ($p<0.001$) or to be on guideline recommended statin intensity ($p< 0.001$).
75	Norberg H. et al.	2020	Sweeden	Prospective cohort	n= 1924 subjects with Heart failure	Men were prescribed significantly higher doses of ACE-I/ARB and MRA. No differences in BBs dose.
78	Perera, S. et al.	2021	Australia	Prospective cohort	n = 729 subjects with Coronary Artery Disease	Dual antiplatelets, statins, ACE-I/ARBs were similar between gender at discharge. After 12-months, statin use was significantly lower in women.
79	Peters, S.A.E. et al.	2018	US	Prospective cohort	n = 88256 subjects with subjects with Myocardial infarction	30 days after the event, there were significant difference in prescription of all evidence-based treatments. Women were less likely than men to fill high-intensity statins, and the disparity was largest in the youngest and oldest adults and for those without prevalent comorbid conditions.
80	Rachamin, Y. et al.	2021	Switzerland	Cross-sectional	n = 2398 Cardiovascular disease	Women were less often treated with lipid-modifying drugs and received less high-intensity treatment than men. No significant difference was found in antihypertensive drugs.
82	Rangaiah, S.K.K. et al.	2020	India	Prospective cohort	n = 621 subjects with Major cardiovascular event	Use of aspirin, clopidogrel and statins was high (>97%) without any gender difference. There were no gender differences in use of ACE-I/ARBs and BBs, but nitrates were used more commonly in women ($p = 0.003$).
83	Redfors, B. et al.	2015	Sweeden	Prospective cohort	n = 48118 subjects with Myocardial Infarction	Women were less likely to receive aspirin, BBs, ACE-I/ARB, other antiplatelets and statins ($p<0.001$) and more likely to be prescribed nitrates ($p<0.001$).
88	Sakata, Y. et al.	2014	Japan	Prospective cohort	n= 4736 subjects with Chronic Heart Failure	Women were less frequently treated with aspirin, BB, and statin, but more frequently with diuretics.
91	Setny, M. et al.	2021	Poland	Prospective cohort	n= 1236 subjects with Acute coronary syndrome	At discharge, women were prescribed statins less frequently ($p<0.001$) and were less likely to receive high-intensity therapy ($p<0.05$). Gender differences were less pronounced during the follow-up visit.

ACE-I: Angiotensin-converting enzyme; ARBs: Angiotensin receptor blockers; MRA: Mineralocorticoid Receptor Antagonists; BBs: beta blockers.

Table 4a. Summary of publications and main results of **Pharmacological treatment: Prescription** (Continue 5)

Ref	Author	Year	Country	Study Design	Sample characteristics	Main findings
92	Shehab A. et al.	2020	Arab emirates	Prospective cohort	n= 31620 subjects with ST elevation Myocardial Infarction	Women were less likely to receive guideline-recommended therapies at discharge than men.
94	Sobers, N. et al.	2019	US	Prospective cohort	n = 1018 subjects with Acute coronary syndrome	Compared with men, a higher proportion of women were discharged on all recommended treatments, only being statistically significant for beta-blockers.
95	Sotorra-Figuerola, G. et al.	2022	Spain	Prospective cohort	n = 8071 subjects with Acute myocardial infarction	More men than women received guideline recommended drugs (antiplatelet agents, statins, and BBs). Non-significant differences were found in treatment with ACEI/ARBs. Comedications were higher in women (anticoagulants, CCB, diuretics), except nitrates. More men were treated with the combination of the 3-4 drugs ($p < 0.001$).
97	Tan YC. et al.	2016	England	Literature review	n= 77 articles about subjects with Acute coronary syndrome	Women were less likely to be discharged on clopidogrel, aspirin, BBs, lipide-modifying agents, such as statins, and ACE-I/ARB ($p<0.001$)
99	Victor, B.M. et al.	2014	US	Retrospective cohort	n = 9950 subjects with Coronary artery disease	Women were less likely to be treated with statins and aspirin, ACE-I/ARB, and BB ($p < 0.05$) and to be on a higher potency statin, lipid-lowering combination, or other lipid-lowering drug ($p <0.001$).
100	Virani, S.S. et al.	2015	US	Prospective cohort	n= 972532 subjects with Cardiovascular disease	Women were less likely than men to receive statins ($p <0.001$) or high-intensity statins ($p <0.001$).
101	Vynckier, P. et al.	2021	Multiple	Prospective cohort	n= 8261 subjects with Coronary event	No significant gender difference in prescription and use of aspirin/ other antiplatelet and BBs.<br>ACE-I were more often prescribed and used in men while ARBs were in women. No differences when both drugs were counted as one entity.<br>Men were more likely to be prescribed and use statins and anticoagulants. Women were more likely to use CCB and diuretics.

BBs: beta blockers; ACE-I: Angiotensin-converting enzyme; ARBs: Angiotensin receptor blockers; CCB: Calcium channel blockers.

Table 4a. Summary of publications and main results of **Pharmacological treatment: Prescription** (Continue 6)

Ref	Author	Year	Country	Study Design	Sample characteristics	Main findings
103	Wei, J. et al.	2017	US	Prospective cohort	n = 4918 subjects with ST Elevation Myocardial infarction	Women were slightly less likely to be discharged on aspirin, statin and other antiplatelet agent compared with men ($p < 0.05$).
104	Wilkinson, C. et al.	2019	England	Prospective cohort	n= 691290 subjects with Myocardial infarction	Women less frequently were discharged on statin, ACE- I/ARB or BB ($p < 0.001$).
105	Xia, S. et al.	2020	China	Cross-sectional	n = 5454 subjects with Cardiovascular disease	Secondary preventive medicine use in women was consistently less frequent than that in men.
20	Yu, B. et al.	2015	China	Clinical trial	n= 9420 subjects with Coronary heart disease	Almost all patients were treated with monotherapy, being atorvastatin and simvastatin the most frequently statins prescribed with no gender differences. Female patients received lower potency level statin dose than men.
106	Zhang, H. et al.	2016	US	Prospective cohort	n = 24338 subjects with Coronary artery disease	Female sex was associated with lower likelihood of statin use. Four factors together accounted 90.4% of the gender disparity in statin therapy: age, smoking, cardiology evaluation and adverse reactions.
108	Zhao M. et al.	2020	Multiple	Meta-analysis	n= 2264600 (43 studies) about subjects with Cardiovascular medication	Women were less likely to be prescribed aspirin, statins, BBs, and ACE-I. They were more likely to be prescribed with diuretics. Gender differences in ratio of prescription did not change over time for aspirin, BBs, ACE-I and diuretics.
109	Zheng, H. et al.	2019	Singapore	Retrospective cohort	n = 7597 subjects with ST elevation Myocardial infarction	Prescription rates of aspirin, and P2Y12 antagonists were lower in women, but there were no significant sex differences in BBs, lipid lowering drugs and ACE-I prescription at discharge.

ACE-I: Angiotensin-converting enzyme; ARBs: Angiotensin receptor blockers; BBs: beta blockers; P2Y12: purinergic receptor P2Y, G-protein coupled, 12 proteins.

Table 4b. Summary of publications and main results of **Pharmacological treatment: Adherence**

Ref	Author	Year	Country	Study Design	Sample characteristics	Main findings
26	Bhuyan, S.S. et al.	2017	US	Cross-sectional	n= 14226 subjects with Cardiovascular disease	Cost-related medication nonadherence behaviours were more likely among women, who more frequently skipped medication, took less or delayed filling.
40	Eindhoven, D.C. et al.	2017	Netherlands	Cross-sectional	n = 59534 subjects with Myocardial infarction	Statistically significant gender differences were found for the achievement of medical adherence, particularly in the use of aspirin and statins.
61	Lauffenburger, J.C. et al.	2014	US	Retrospective cohort	n = 85017 subjects with Acute myocardial Infarction	1 year after AMI, Black and Hispanic women had the lowest likelihood of being adherent, followed by white and Asian, and black and Hispanic men.
67	M.T. Gürgöze, et al.	2021	Netherlands	Cross-sectional	n= 25776 subjects with Chronic Heart failure	Adherence was similar in men and women. Statistically significant difference was observed for women using ACE- I/ARBs, diuretics and MRAs. Men were less adherent to BBs.
71	Moaddab, F. et al.	2023	Iran	Cross-sectional	n= 115 subjects with Heart failure	There was no significant relationship between medication adherence and gender.
74	Nanna MG. et al.	2019	US	Cross-sectional	n = 5693 subjects with Guideline indication for statin treatment	Women were more likely to report never being offered, discontinuing or declining statin treatment ($p < 0.001$).
91	Setny, M. et al.	2021	Poland	Prospective cohort	n= 1236 subjects with Acute coronary syndrome	No statistically significant gender differences in terms of discontinuation or reduction of the statin dose. Statins were more used if patients visited a cardiologist.
101	Vynckier, P et al.	2021	Multiple	Prospective cohort	n= 8261 subjects with Coronary event	Analyses on medication adherence showed no gender differences in the adherence to antihypertensive, lipid and glucose-lowering drugs.

AMI: Acute myocardial infarction; ACE-I: Angiotensin-converting enzyme; ARBs: Angiotensin receptor blockers; MRA: Mineralocorticoid Receptor Antagonists; BBs: beta blockers.
